# Case series: Nocardiosis of the brain and lungs

**DOI:** 10.4103/0971-3026.41830

**Published:** 2008-08

**Authors:** Seema Sud, TBS Buxi, Ish Anand, Anshu Rohatgi

**Affiliations:** Department of Radiology, Sir Ganga Ram Hospital, Rajinder Nagar, New Delhi - 110 060, India; 1 Department of Neurology, Sir Ganga Ram Hospital, Rajinder Nagar, New Delhi - 110 060, India

**Keywords:** CT, MRI, nocardiosis

## Abstract

Localized and multisystem nocardiosis is an opportunistic disease that occurs commonly in immunocompromised patients. Rarely, it is also seen in immunocompetent individuals. The lungs and brain are commonly involved. Typical, but nonspecific, findings are often seen on imaging and the presence of concomitant lesions in these two systems often suggests this diagnosis. We report two cases of cerebral and pulmonary involvement by nocardiosis.

## Case 1

A 70-year-old man, a known case of idiopathic thrombocytopenic purpura (ITP), on a 20-mg daily dose of steroids for the last 3 years, presented with sudden-onset slurring of speech that progressed over the next 24 h to a complete inability to speak. Within a week, the patient developed difficulty in swallowing both solids and liquids, along with a choking sensation and an inability to protrude his tongue. He also had two episodes of generalized tonic-clonic seizures. There was no history of fever, headache, vomiting, or loss of conciousness. On examination the patient was well oriented in time and space and his vital parameters were maintained. There was generalized wasting of the muscles, with bilateral ptosis and facial palsy. Power was grade 4/5 in all the limbs and the plantars showed bilateral withdrawal.

The laboratory investigations revealed a raised white blood cell count and reduced platelet count, total proteins, and albumin. The blood urea nitrogen, creatinine, random blood sugar, serum electrolytes, and liver function tests were within normal limits. The chest radiograph was also normal.

**Figure 1 (A, B):**
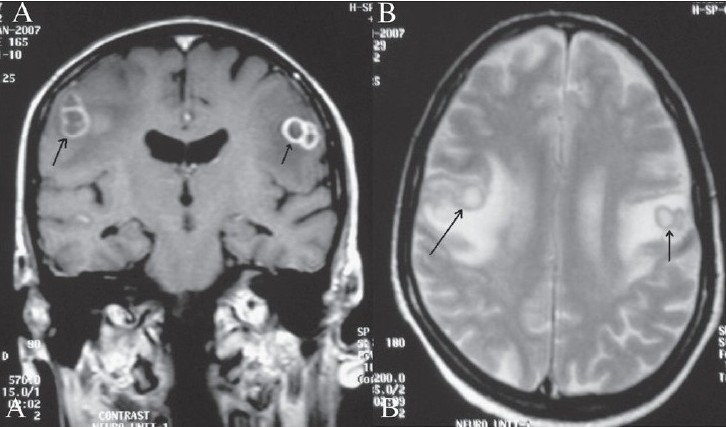
Contrast T1W coronal (A) and axial T2W MRI images of the brain show conglomerate ring-enhancing lesions in both the frontal subcortical regions (arrows in A), with central hyperintensity and peripheral low signal intensity rims (arrows in B)

MRI of the brain revealed well-defined ring-enhancing lesions in both the frontal subcortical regions [Figure 1A], with significant surrounding edema. The lesions displayed central hyperintensity, with a peripheral low signal intensity rim on the T2W images [Figure 1B]. On T1W images, the central contents were hypointense and the walls showed intermediate signal intensity. Similar lesions were also seen in both occipital regions and the right temporal region. Minimal meningeal enhancement was also seen in the right occipital and high frontoparietal regions bilaterally. A diagnosis of multiple brain abscesses was made.

**Figure 2:**
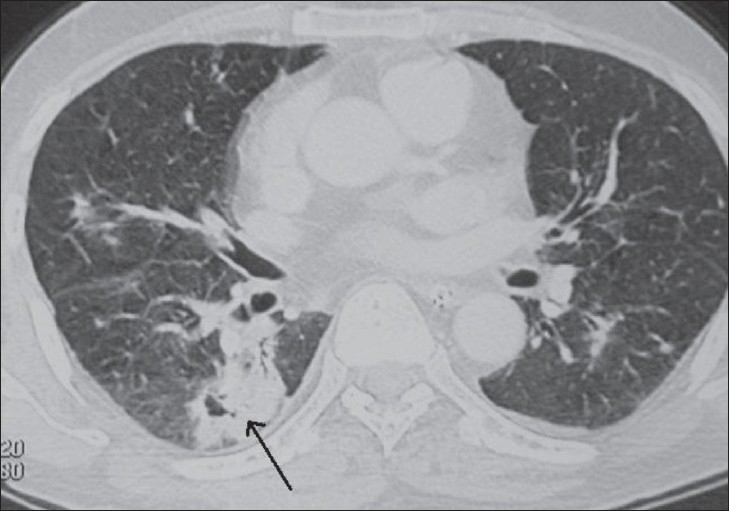
CT scan of the chest shows consolidation and collapse with internal breakdown in the superior segment of the right lower lobe (arrow)

CT scan of the thorax [Figure 2] revealed consolidation and collapse with necrosis in the superior segment of the right upper lobe. Small areas of alveolar opacification were seen in both the lungs. A small left pleural effusion and a pericardial effusion were also seen.

**Figure 3 (A, B):**
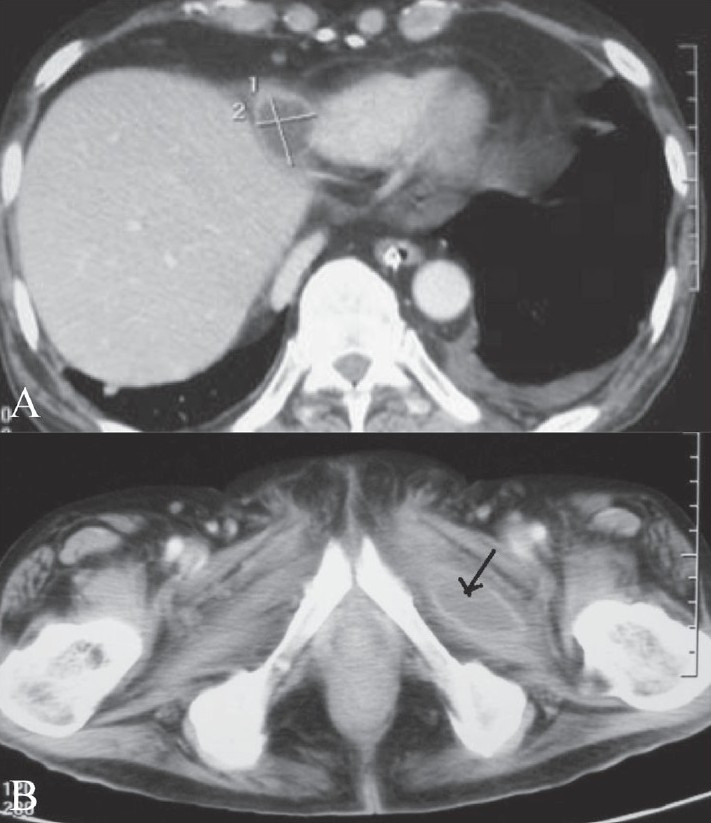
Along with an abscess in the right paracardiac region (A) CT scan of the abdomen shows an abscess (arrow) in the left obturator externus muscle (B)

CT scan of the abdomen [Figure 3] revealed multiple loculated abscesses within the muscular planes of the thoracoabdominal wall as well as in the left parapsoas region and the right iliacus, left obturator externus, and posterior spinal muscles. Collections were also seen within the mesentry and in the right paracardiac region.

CT-guided aspiration from the abscess in the posterior spinal muscle was performed; the aspirate grew Nocardia on culture. Pus aspirated from the intracranial lesion under local anesthesia also grew nocardia. The organism was identified upto the genus level; however, speciation could not be done.

The patient was put on appropriate treatment.

## Case 2

A 60-year-old man who had had diabetes and hypertension for the last 15 years presented with a history of right-sided weakness which progressed slowly over a period of 2 weeks. A week before presentation he had also developed headache, vomiting, and difficulty in swallowing. At presentation, he had loss of speech followed by altered sensorium. The patient had also been having cough with minimal sputum off and on and loss of weight for the last 3 months.

The patient had a focal lesion on a brain MRI, along with a left pleural effusion on a chest radiograph, and had been treated for tuberculosis. When he was finally seen in our hospital, the patient was afebrile and had a raised pulse rate and blood pressure. The liver was mildly enlarged. Respiratory and cardiovascular system examination was unremarkable. He had altered sensorium, with aphasia and complete weakness of the right side of the body (grade 0/5 power in both upper and lower limbs). The plantar was upgoing on the right side and there was gaze preference to the left. Fundus examination revealed a few microaneurysms at the macula, a few cotton wool spots, and mild arteriolar attenuation in both the eyes.

The laboratory examination revealed normal red blood cell and renal function parameters, with normal sodium and potassium levels. The total leucocyte count was reduced.

**Figure 4 (A-C):**
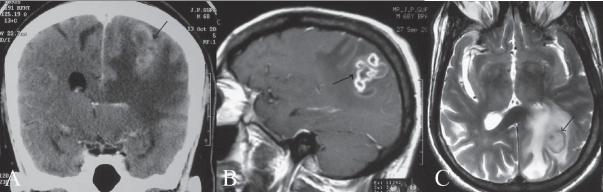
Coronal contrast-enhanced CT (A), contrast-enhanced sagittal MRI (B), and T2W axial MRI (C) images of the brain show conglomerate ring-enhancing lesions in the left parietal lobe, with significant surrounding edema and contralateral shift (arrows in A and B). Note the central hyperintense signal with low signal intensity walls (arrow in C)

**Figure 5 (A, B):**
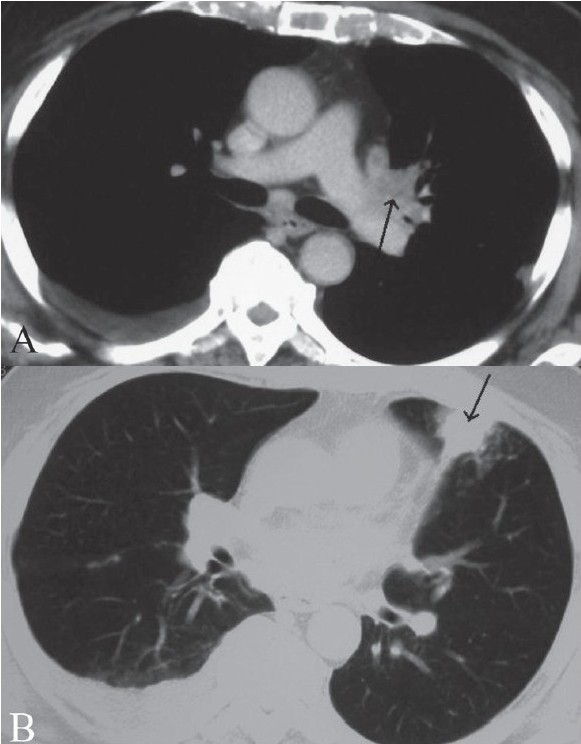
Soft tissue (A) and lung window (B), contrast-enhanced CT images of the chest show necrotic lymphnodes in the left hilar region (arrow) with consolidation in the left lung (arrow)

Contrast-enhanced CT of the head [Figure 4A] revealed conglomerate, ring-enhancing lesions in the left parietal lobe, with significant surrounding edema and contralateral shift. There was no abnormal meningeal enhancement. Contrast-enhanced MRI of the brain revealed perpherally enhancing conglomerate lesions in the left parietal lobe [Figure 4B], with significant surrounding edema. These lesions showed central hyperintense signals with low signal intensity walls on the T2W images [Figure 4C]. On T1W images, the central contents appeared hypointense. USG of the abdomen showed a small right pleural effusion and hepatomegaly. Contrast-enhanced CT scan of the chest revealed necrotic lymph nodes in the left hilar region [Figure 5A] and consolidation in the left lung [Figure 5B]. Bronchial aspiration did not reveal the presence of acid fast bacilli (AFB). Fungal smear was also negative.

The patient showed no response to antituberculous treatment. A temporoparietal craniotomy was performed and the cyst was excised. The histopathology revealed nocardiosis, which was identified up to the genus level; speciation was not done.

## Discussion

Nocardiosis, caused by gram-positive, weakly acid-fast, filamentous aerobic actinomycetes, is an opportunistic infection. *Nocardia asteroides* is the most common species that infects humans. An increasing number of cases are being reported in immunocompetent individuals without predisposing factors.[1]

Involvement of the lungs (75-80%) and the skin is the most common form of presentation, but virtually any organ system may be involved.[2–4] The central nervous system is involved in approximately 44% of cases, usually in patients having pulmonary infection, as seen in both our cases.[5] When there is involvement of two or more noncontiguous organs, with or without CNS involvement, the disease is said to be disseminated. The incidence of disseminated disease is 25-40%.[6] Both our cases had disseminated disease.

Imaging findings are not specific for nocardiosis. In the chest, it can present as pulmonary consolidation, irregular nodular parenchymal densities, solitary irregular lung mass, interstitial reticular pattern, pleural effusion, or lymphadenopathy, but none of these is a specific diagnostic feature.[7] Both our patients had areas of consolidation in the lungs.

The imaging findings in the brain depend on the stage of the infectious process at the time of imaging; this may vary from cerebritis to frank abscess formation. In the abscess stage, the necrotic debris accumulates centrally, while the collagenous capsule is being formed. The proteinaceous, necrotic debris has signal intensity higher than that of the CSF on T1W and turbo inversion-recovery (IR) images, with a moderate degree of brain edema. On T1W images, the abscess capsule stands out against the necrotic center and surrounding edema as an isointense to slightly hyperintense ring. On T2W images, the ring is consistently hypointense. This hyperintense capsule may either be due to capsular hemorrhage, the paramagnetic methemoglobin causing increased signal on T1W images, or due to an abundance of free radicals in the macrophages in the abscess capsule. The activity of the macrophages is highest in the late cerebritis and early abscess phases, at which time the capsule shows marked hypointensity on T2W images.[8] Ring enhancement is seen, which may persist for upto 8 months after treatment and therefore should not be interpreted as failure of treatment. Reliable signs of good response to treatment are shrinkage of the necrotic center and decrease in capsular hypointensity on T2W images.

The diagnosis is confirmed by direct microscopy and culture.[1] Demonstration of marked polymorphonuclear leukocytes in the pus, in the absence of regional lymph node enlargement, is said to be characteristic.[6]

There are only three previous reports on disseminated nocardiosis in patients with chronic ITP.[2,3,6] These patients need to be treated with intensive combination chemotherapy, along with slow tapering off of the steroids.[9–11] The drug of choice is co-trimoxazole[3,4] which can be given alone or in combination with one or more of the following antimicrobial drugs: imipenim, amikacin, ampicillin, third-generation cephalosporins, fluoroquinolones, or minocylines; co-trimoxazole is usually given by the intravenous route in cerebral nocardiosis. The usually recommended duration of therapy is for 6-12 months.[3] The decision to intervene surgically varies from case to case.[3]

Our first patient, who was a known case of ITP and had disseminated nocardial infection, showed initial improvement in the skin and lung lesions, but there was no improvement in the cerebral lesions. As there was subsequent neurological deterioration, the right frontal brain abscess was surgically excised under intensive platelet cover. He initially showed improvement but eventually succumbed to his illness after 4 months of treatment.

Our second patient underwent a temporoparietal craniotomy, with excision of the abscess, as he was not responding to antituberculous treatment and a histopathological diagnosis had to be obtained. He did well postoperatively for a period of one month, but then he too succumbed to his illness.

An overall mortality of 40-60% has been reported in disseminated nocardiosis.[4]

When patients present with comcomitant brain and lung lesions, the differential diagnoses to be kept in mind include tuberculosis, neoplastic disease, and nocardiosis. Nocardiosis can affect both immunocompromised and immunocompetent individuals. As the imaging findings are nonspecific and the causative organism is difficult to isolate, diagnosis is often a problem and hence it is important to have a high index of suspicion in the appropriate clinical settings, especially in patients not responding to antituberulous treatment. Skin involvement occurring along with lung and brain lesions is commonly seen in nocardiosis and is a pointer towards the diagnosis. Multidrug medical therapy, with surgical drainage of the abscess, is advocated in the disseminated form of the disease.
